# Global Surgeon Opinion on the Impact of Surgical Access When Using Endocutters Across Specialties

**DOI:** 10.36469/001c.87644

**Published:** 2023-09-20

**Authors:** Marina Gutierrez, Nadine Jamous, William Petraiuolo, Sanjoy Roy

**Affiliations:** 1 Johnson & Johnson MedTech, New Brunswick, New Jersey, USA

**Keywords:** endocutter, modified Delphi panel, surgical access

## Abstract

**Background:** Despite design enhancements in endocutters, key challenges related to limited surgical access and space can impact stapling and, potentially, surgical outcomes. **Objectives:** This study aimed to develop consensus statements outlining the clinical value of precise articulation and greater anatomical access in minimally invasive surgery performed by bariatric, colorectal, and thoracic surgeons. **Methods:** Colorectal, bariatric, and thoracic surgeons from Japan, the United States, United Kingdom, and France participated in a 2-round modified Delphi panel. Round 1 included binary, Likert scale–type, multiple-response, and open-ended questions. These were converted to affirmative statements for round 2 if sufficient agreement was reached. Consensus was set at a predefined threshold of at least 90% of panelists across all surgical specialties and regions selecting the same option (“agree” or “disagree”) for the affirmative statements. **Results:** Of the 49 statements in the round 2 questionnaire, panelists (n=135) reached consensus that (1) tissue slippage outside stapler jaws can occur due to limited access and space; (2) greater jaw aperture could help to manipulate thick or fragile tissue more easily; (3) articulation of an endocutter is clinically important in laparoscopic surgeries; (4) improved access to hard-to-reach targets and in limited space would improve safety; and (5) an endocutter with improved access through greater articulation would become common use. **Discussion:** By understanding user-specific challenges and needs from both specialty- and region-wide perspectives, endoscopic stapling devices can continue to be refined. In this study, improved articulation and greater jaw aperture were the key design features examined. Improved articulation and greater jaw aperture were key stapler design features identified in this study that may mitigate the risk of instrument clashes and intraoperative complications such as anastomotic leaks. **Conclusions:** This study gained insights into surgeons’ perspective across a variety of specialties and from 3 distinct geographies. Participating surgeons reached consensus that an endocutter with greater jaw aperture and articulation may improve surgical access and has potential to improve surgical outcomes.

## INTRODUCTION

There has been widespread adoption of minimally invasive surgery (MIS), which includes laparoscopic and thoracoscopic procedures, both globally and across surgical specialties.^1,2^ The International Federation for the Surgery of Obesity and Metabolic Disorders (IFSO) estimated that between 99.2% and 99.7% of bariatric surgeries were performed laparoscopically among its 50 contributor countries between 2016 and 2020.^3^ Similarly, the use of video-assisted thoracoscopic surgery (VATS) has been increasing worldwide and is the most common approach for lung cancer resection in the United Kingdom (UK).^4^ A 2012 analysis estimated that 43% of colorectal procedures in the United States (US) were minimally invasive.^5^ MIS can result in faster recovery times, reduced postoperative pain, shortened hospital stays, and improved cosmesis compared with open surgery.^6–9^

Laparoscopic surgical stapling devices facilitate tissue approximation and transection during MIS. Studies comparing stapling devices against suturing found that similar operative outcomes were achieved.^10,11^ Given the variable dynamic characteristics of tissue, including thickness, compressibility, and elasticity, surgical devices need to complement target tissue properties for optimal stapling.^12^ Adequate grip is also key to ensure staple alignment during firing by countering potential tissue slippage.^13^ Powered staplers (ie, those for which the staples and the knife blade are driven not by manual force but instead by a battery) provide several benefits compared with non-powered (manual) devices.^14^ By reducing the manual force expended by surgeons, their energy can be diverted to maintaining stability and enable more precise and reliable stapling. Relative to manual devices, powered staplers may be associated with improved clinical outcomes and decreased hospital costs.^14–18^

Despite the advancement in endoscopic staplers, key clinical challenges related to limited surgical access and space can impact acceptable stapling and surgical outcomes. Tight spaces in areas like the pelvis and chest cavity can be challenging for surgeons to maneuver and optimally position the stapler.^12^ Thicker tissue such as lung parenchyma and stomach can be difficult to capture and are more susceptible to tissue slippage, therefore potentially requiring additional manipulation.^12,19^ The ability to place the jaws of the stapler precisely where the surgeon intends is key to enabling surgeons to successfully carry out procedures.

We sought to understand the potential impact and unmet need, from the surgeons’ perspective, of limited surgical access and space for stapling, which to our knowledge have not been documented. The objective of this study was to develop consensus statements outlining the clinical value of precise articulation and greater anatomical access in MIS procedures performed by bariatric, colorectal, and thoracic surgeons.

## METHODS

The modified Delphi method is a widely used, systematic, and robust methodology to gather expert consensus using several rounds of iterative questionnaires.^20,21^ Participants are provided with a series of questions to answer anonymously, eliminating any influence of group pressure on individuals’ responses. The Delphi panel process enables users to share their experiences, needs, and challenges in an open-ended format, such that the impact of stapling device design can be evaluated and potentially utilized to facilitate design improvements to address any unmet need(s).

### Delphi Panelists

Eligible panelists from Japan, the US, the UK, and France were board-certified colorectal, bariatric, or thoracic full-time practicing surgeons who perform on average at least 10 relevant surgeries per month. In addition, panelists must have performed at least 10 procedures per month using endocutters (endoscopic linear stapling device). Panelists were invited via email and asked to respond in the affirmative if they wished to participate in the Delphi panel. Complete eligibility criteria are available in **Supplementary Table S1**.

### Study Procedures and Evaluations

This study used a modified Delphi method, which prespecified that 2 questionnaire rounds would be conducted and delivered through a web-based platform. The first round included a screening questionnaire to ensure all potential panelists met the eligibility criteria. Questions in round 1 were developed from assumptions of the unmet need, based on current literature, and the authors’ own subject matter expertise and experience. A detailed list of the round 1 and 2 survey questions is available in the **Supplementary Material**.

The question types posed in the survey were open-ended, ranking, multiple-response, binary (yes/no and agree/disagree) and Likert scale. Panelists were able to provide a free-text comment to contextualize their response and/or make suggestions for round 2.

Responses to the Likert scale questions were given on a 5-point scale where 5 = extremely clinically important, 4 = very clinically important, 3 = somewhat clinically important, 2 = not very clinically important, and 1 = not at all clinically important. Responses from round 1 facilitated the development of binary-response questions for round 2. Statements from round 1 that had at least 70% agreement among respondents in all counties or specialties and/or were ranked in the top 3 most common responses (for multiple-response questions or ranking questions) were further explored in round 2, where respondents had the option to agree or disagree with the most common responses presented as affirmative statements. All responses provided during the Delphi rounds were anonymized. No patients were involved in the study, and therefore ethical approval was not required. Respondents and study sponsors were blinded to each other.

### Statistical Analysis

Results were exported directly from the online survey and analyzed in Microsoft Excel. Consensus was set at a predefined threshold of at least 90% of panelists across every surgical specialty (ie, bariatric, thoracic, colorectal) and region (ie, US, Europe, and Japan) selecting the same option (agree or disagree) for the affirmative statements posed in round 2. Agreement rather than consensus was established by at least 90% of panelists within a subgroup (ie, either by specialty or region) selecting the same option (either agree or disagree). Responses from surgeons in France and UK were grouped together under the broader category of Europe. The predefined ≥90% threshold was chosen to increase fidelity and ensure that consensus could be achieved within 2 rounds, as is conventional in a modified Delphi study. It has been noted that the definition of consensus used in published Delphi studies is discrepant, with thresholds varying widely from 50% to 97%.^21^

## RESULTS

**Figure 1.**
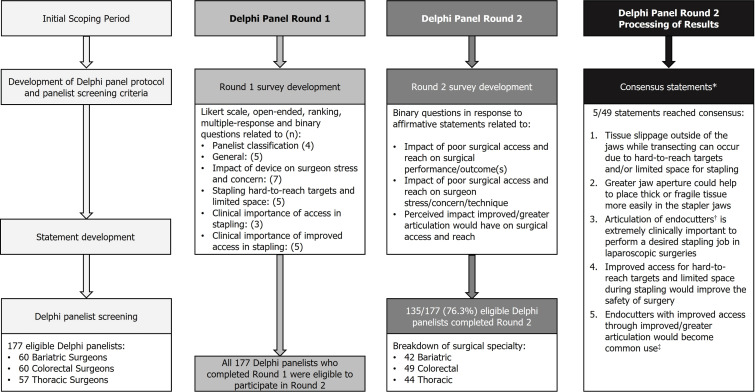
Delphi Panel Study Flowchart *Consensus was set at a predefined threshold of at least 90% of panelists across every surgical specialty (ie, bariatric, thoracic, colorectal) and region (ie, US, Europe, and Japan) selecting the same option (either “agree” or “disagree”) for the affirmative statements posed in round 2. †“Endoscopic linear stapler/stapling device” was used in the Delphi panel questionnaire to refer to “endocutters,” and these two terms have been used interchangeably. ‡“Standard of care” rather than “common use” was presented in the Delphi panel questionnaire.

Round 1 of the Delphi panel was conducted from March 23, 2022, to April 29, 2022. Round 2 was open from August 12, 2022, to September 22, 2022. In total, 177 eligible panelists completed round 1, with a total of 135 panelists having completed both round 1 and 2 questionnaires (**Figure 1**).

Of the 135 panelists that completed round 2, 31.1% were bariatric surgeons, 32.6% thoracic surgeons, and the remaining 36.3% colorectal surgeons. The majority of respondents were male (94.1%) and performed the majority of surgeries laparoscopically (for bariatric and colorectal surgeons, 53.7%) or as VATS (for thoracic surgeons, 50.0%). A description of panelists who completed rounds 1 and 2 is presented in **Table 1**.

**Table 1.** </strong> Delphi Panelist Characteristics of Those Who Completed Rounds 1 and 2

**Characteristic**	**Value, n (%)**
Panelists	135
Male	127 (94.1)
Female	8 (5.9)
Bariatric surgeons	42 (31.1)
Average No. (proportion) of laparoscopic procedures per surgeon/mo	20 (51.0)
Colorectal surgeons	49 (36.3)
Average No. (proportion) of laparoscopic procedures per surgeon/mo	28 (55.9)
Thoracic surgeons	44 (32.6)
Average No. (proportion) of VATs per surgeon/mo	14 (50.0)
Practice setting	
Teaching hospital (public or private)	73 (54.1)
Non-teaching hospital	55 (40.7)
Others	7 (5.2)
Regions represented	
Europe^a^	40 (29.6)
France	19 (14.1)
UK	21 (15.6)
Japan	55 (40.7)
US	40 (29.6)

Abbreviation: VATS, video-assisted thoracoscopic surgery.

^a^ Percentage calculated using the number of panelists from France and UK (n = 40) and not summing individual percentage values.

The affirmative statements presented in round 2 were divided into 3 main areas: impact of limited surgical access and space on surgical performance/outcome(s) (**Table 2**), impact of limited surgical access and space on surgeon stress/concern/technique (**Table 3**), and perceived impact of improved/greater articulation in stapling on surgical access (**Table 4**). Overall, 49 statements were developed from responses provided in round 1. Of the 49 statements in the round 2 questionnaire, 5 statements reached consensus: 1 relating to the impact of limited surgical access and space on surgical performance/outcome(s) (consensus statement 1) and 4 on the perceived impact of improved/greater articulation in stapling on surgical access (consensus statements 2-5):

1. Tissue slippage outside of the jaws while transecting can occur due to hard-to-reach targets and/or limited space for stapling.
2. Greater jaw aperture feature could help to place thick or fragile tissue more easily in the stapler jaws.
3. Articulation of endocutters is extremely clinically important to perform a desired stapling job in laparoscopic surgeries.
4. Improved access for hard-to-reach targets and limited space during stapling would improve safety of surgery.
5. Endocutters with improved access through improved/greater articulation would become common use.^22^

**Table 2.** Survey Results from Round 2 on the Impact of Limited Surgical Access and Space on Surgical Performance/Outcome(s)

Statement	Consensus Agreement^a^	Percentage Agreement						
		Total	Region			Specialty		
			US	Europe	Japan	Bariatric	Colorectal	Thoracic
Impact of limited surgical access and space when using endocutters^b^								
Tissue slippage outside of the jaws while transecting	Consensus agreement reached	92%	90%	95%	91%	95%	90%	91%
Increased tension on the structure or tissue surgeon is firing on	Agreement reached in 4/6 subgroups	92%	88%	98%	91%	88%	94%	93%
Needing extra reloads to compensate for tissue slipping outside the jaws	Agreement reached in 2/6 subgroups	88%	95%	88%	84%	83%	96%	84%
Tearing of fragile tissue away from the staple line	Agreement reached in 2/6 subgroups	88%	85%	90%	89%	86%	92%	86%
Tearing of fragile tissue along the staple line	Agreement reached in 2/6 subgroups	86%	78%	95%	85%	86%	94%	77%
Poor staple line quality	Agreement reached in 2/6 subgroups	84%	78%	90%	84%	86%	92%	73%
Complications of limited surgical access and space when using endocutters to create staple lines								
Less surgical margin than expected	Agreement reached in 2/6 subgroups	89%	93%	88%	87%	86%	94%	86%
Tissue trauma that requires further repair	Agreement reached in 3/6 subgroups	89%	95%	95%	80%	88%	92%	86%
Staple line oozing/bleeding controllable with clips, suture, or fibrin glue	No agreement reached in any subgroup	85%	83%	83%	89%	81%	88%	83%
Tearing of fragile tissue away from the staple line	Agreement reached in 1/6 subgroups	84%	78%	85%	87%	86%	90%	75%
Tearing of fragile tissue along the staple line	Agreement reached in 2/6 subgroups	86%	80%	90%	87%	86%	92%	80%

Shaded values indicate agreement.

^a^Consensus was set at a pre-defined threshold of ≥90% of panelists across every surgical specialty (ie, bariatric, thoracic, colorectal) and region (ie, US, Europe and Japan) selecting the same option (either “agree” or “disagree”) for the affirmative statements posed in round 2.

^b^“Endoscopic linear stapler/stapling device” was used in the Delphi panel questionnaire to refer to “endocutters,” and these two terms have been used interchangeably.

**Table 3.** Survey Results from Round 2 on the Impact of Limited Surgical Access and Space on Surgeon Stress/Concern/Technique

**Statement**	**Consensus Agreement^a^**	**Percentage Agreement**						
		**Total**	**Region**			**Specialty**		
			**US**	**Europe**	**Japan**	**Bariatric**	**Colorectal**	**Thoracic**
**Type of compensating behavior adopted to and/or adjust for in hard-to-reach targets and limited space**								
Angulation change/change angle of approach/twist/rotate/move around	Agreement reached in 4/6 subgroups	93%	95%	100%	85%	88%	98%	91%
Visualization change/look from different perspectives	Agreement reached in 4/6 subgroups	90%	90%	93%	89%	88%	92%	91%
Additional/further dissection	Agreement reached in 2/6 subgroups	87%	90%	85%	87%	88%	90%	84%
Adding or using another port	Agreement reached in 1/6 subgroups	84%	78%	75%	95%	88%	86%	77%
Delicate/slower/more fine handling of stapler	Agreement reached in 2/6 subgroups	84%	78%	80%	91%	93%	80%	80%
Suturing over trouble spots	No agreement reached in any subgroup	81%	80%	73%	87%	83%	82%	77%
The use of additional trocars/switch or change trocars	Agreement reached in 1/6 subgroups	79%	70%	70%	91%	83%	80%	73%
The use of different staples/different staple loads	Agreement reached in 1/6 subgroups	76%	63%	78%	85%	64%	90%	73%
The use of multiple fires/double stapling	No agreement reached in any subgroup	76%	73%	65%	87%	76%	82%	70%
The use of staple line reinforcing	No agreement reached in any subgroup	71%	65%	65%	80%	74%	78%	61%
**Stress or concern experienced by surgeons**								
Anticipate that the surgeon would have less stress or concern if the surgeon was using endocutters^b^ with improved articulation in surgery	Agreement reached in 4/6 subgroups	93%	100%	80%	98%	93%	98%	89%
Anticipate that the surgeon would have less stress or concern if the surgeon was training a fellow or resident during surgery with endocutters with improved articulation	Agreement reached in only 1/6 subgroups	79%	73%	68%	91%	74%	88%	73%
Experience stress or concern when the surgeon has a less accessible approach to a vessel or pedicle that may increase the chance of harm/injury to a vessel or pedicle	Agreement reached in 3/6 subgroups	84%	93%	65%	93%	90%	78%	86%
Experience stress or concern that a hard-to-reach bleeding pedicle or vessel may lead to a higher probability of surgical complication	No agreement reached in any subgroup	81%	85%	80%	80%	79%	86%	80%
Experience stress or concern when the surgeon must adjust to the limits of the device	No agreement reached in any subgroup	79%	83%	60%	89%	88%	80%	68%
Experience stress or concern when the surgeon needs to convert from laparoscopic to open surgery due to failure of endocutters to secure pedicle or blood vessel	No agreement reached in any subgroup	79%	78%	68%	87%	86%	73%	77%
Do not experience stress or concern that a hard-to-reach area may compromise my surgical training during a procedure	No agreement reached in any subgroup	39%	30%	40%	44%	36%	41%	39%
Do not experience physical distress or discomfort when facing a situation of a hard-to-reach area with difficult approach to address a bleeding vessel or pedicle	No agreement reached in any subgroup	36%	28%	30%	47%	43%	31%	36%

Shaded values indicate agreement.

^a^Consensus was set at a predefined threshold of at least 90% of panelists across every surgical specialty (ie, bariatric, thoracic, colorectal) and region (ie, US, Europe, and Japan) selecting the same option (either “agree” or “disagree”) for the affirmative statements posed in round 2.

^b^“Endoscopic linear stapler/stapling device” was used in the Delphi panel questionnaire to refer to “endocutters,” and these 2 terms have been used interchangeably.

**Table 4.** Survey Results from Round 2 on Perceived Impact Improved/Greater Articulation in Stapling Would Have on Surgical Access

**Statement**	**Consensus Agreement^a^**	**Percentage Agreement**						
		**Total**	**Regions**			**Specialty**		
			**US**	**Europe**	**Japan**	**Bariatric**	**Colorectal**	**Thoracic**
**Impact of devices with improved/greater articulation in stapling**								
Difficult angles become less stressful	No agreement reached in any subgroup	79%	80%	70%	85%	79%	86%	73%
Space restrictions become less stressful	No agreement reached in any subgroup	76%	78%	60%	85%	79%	78%	70%
Inability of the device to access target anatomy becomes less stressful	No agreement reached in any subgroup	61%	75%	58%	53%	57%	61%	64%
Decreased visibility to access target anatomy becomes less stressful	No agreement reached in any subgroup	61%	75%	55%	55%	55%	67%	59%
Features that facilitate reaching difficult-to-access targets								
Greater jaw aperture feature could help to place thick or fragile tissue more easily in the stapler jaws	Consensus agreement reached	93%	95%	95%	91%	90%	90%	100%
Greater articulation span feature could help the surgeon reach difficult to access targets	Agreement reached in 3/6 subgroups	90%	93%	85%	91%	86%	94%	89%
Greater jaw aperture feature could help the surgeon reach difficult to access targets	Agreement reached in 2/6 subgroups	87%	80%	85%	95%	83%	90%	89%
Easy to use, one-handed operation feature could help the surgeon reach difficult to access targets	Agreement reached in 1/6 subgroups	84%	83%	88%	84%	74%	92%	86%
Important concerns/considerations for laparoscopic surgeons								
When performing a desired stapling job, the articulation of endocutters^b^ is extremely clinically important for laparoscopic surgeries	Consensus agreement reached	93%	93%	90%	96%	95%	92%	93%
Surgeons perceive that improved access through improved/greater articulation in stapling would have a positive clinical effect on surgical outcomes	Agreement reached in 2/6 subgroups	87%	98%	83%	84%	79%	94%	89%
Surgeons have less stress or concern when an assistant is firing endocutters with improved articulation during a robot-assisted surgery procedure, where there may be hard to reach targets and limited space increasing the difficulty of firing	Agreement reached in only 1/6 subgroups	77%	73%	60%	93%	74%	78%	80%
Surgeons spend 10% extra time in pre-operative assessment for patients with predictable compromised surgical access (hard-to-reach targets and limited space)	No agreement reached in any subgroup	65%	53%	60%	78%	67%	67%	61%
Surgeons do not spend extra time in pre-operative assessment for a procedure with predictable compromised surgical access (hard-to-reach targets and limited space)	No agreement reached in any subgroup	44%	35%	38%	55%	40%	47%	43%
**Impact of devices with improved access for hard-to-reach targets and limited space in stapling**								
Improve safety of surgery	Consensus agreement reached	97%	98%	98%	96%	95%	98%	98%
Reduce unintentional tissue/structure damage	Agreement reached in 5/6 subgroups	93%	93%	98%	91%	83%	96%	100%
Reduce tension on the tissue/structure the surgeon was firing on	Agreement reached in 5/6 subgroups	93%	93%	93%	95%	98%	88%	95%
Reduce tearing of fragile tissue away from the staple line	Agreement reached in 3/6 subgroups	91%	88%	88%	96%	93%	88%	93%
Reduce surgical stress	Agreement reached in 4/6 subgroups	89%	90%	83%	93%	83%	92%	91%
**Impact of devices with improved access through improved/greater articulation in stapling**								
Endocutters with improved access through improved/greater articulation in stapling would become common use^c^	Consensus agreement reached	96%	93%	95%	100%	95%	98%	95%
If an endocutter device gave improved access through improved/greater articulation in stapling compared to currently available choices, the surgeons would use in most of their procedures	Agreement reached in 5/6 subgroups	92%	95%	98%	85%	95%	92%	89%

Shaded values indicate agreement.

^a^Consensus was set at a pre-defined threshold of ≥90% of panelists across every surgical specialty (ie, bariatric, thoracic, colorectal) and region (ie, US, Europe, and Japan) selecting the same option (either “agree” or “disagree”) for the affirmative statements posed in round 2.

^b^“Endoscopic linear stapler/stapling device” was used in the Delphi panel questionnaire to refer to “endocutters,” and these two terms have been used interchangeably.

^c^“Standard of care” rather than “common use” was presented in the Delphi panel questionnaire.

### Impact of Limited Surgical Access and Space on Surgical Performance/Outcome(s)

Full results for statements related to the impact of limited surgical access and space on surgical performance/outcome(s) are shown in **Table 2.** Consensus was reached that hard-to-reach targets and/or limited space for stapling can result in tissue slippage outside of jaws while transecting (consensus statement 1), and 4 of 6 subgroups were in agreement that this would also result in increased tension on the structure or tissue the surgeon is firing on; surgeons in the US and bariatric surgeons reached 88% agreement.

**Specialty-specific differences.** Colorectal surgeons reached agreement on all 6 statements relating to the impact of limited surgical access and space on stapling, while thoracic and bariatric surgeons reached agreement on 2 and 1 of these statements, respectively (**Table 2**).

Similarly, colorectal surgeons reached agreement on 4 out of the 5 statements related to complications of limited surgical access and space when using endocutters to create staple lines (**Table 2**). Neither bariatric or thoracic surgeons reached agreement on any of the 5 statements. Across all 11 statements, the minimum level of agreement across surgical specialty was 73%.

**Region-specific differences.** There was most agreement among European surgeons on the impact of limited surgical access and space on stapling (agreement reached on 5 of 6 statements). Surgeons in both the US and Japan reached agreement on 2 of these 6 statements (**Table 2**).

Generally, statements on complications reached less agreement than statements relating to impact of limited surgical access and space. Surgeons in both the US and Europe reached agreement on 2 of 5 statements on the complications, but surgeons in Japan did not reach agreement on any of the statements (**Table 2**). Across all 11 statements, the minimum level of agreement across regions was 78%.

### Impact of Limited Surgical Access and Space on Surgeon Stress/Concern/Technique

Full results of agreement on statements related to the impact of limited surgical access and space on surgeon stress/concern/technique can be found in **Table 3**.

Although none of the statements reached consensus, all surgeons with the exception of those practicing in Japan (85% agreement) and bariatric surgeons (88% agreement) reached agreement that “angulation change/change angle of approach/twist/rotate/move around” was a type of compensating behavior used to adjust for hard-to-reach targets and limited space. In addition, all surgeons except for bariatric surgeons (88% agreement) and those practicing in Japan (89% agreement) reached agreement that having a “visualization change/look from different perspectives” was another form of compensating behavior (**Table 3**).

None of the subgroups reached agreement on whether they experienced stress or were concerned that “hard-to-reach areas [would] compromise their surgical training during a procedure” (range of agreement, 30%-44%). The panelists, within any subgroup, were also not in agreement that they experienced “physical distress or discomfort when facing a situation of a hard-to-reach area [that required a] difficult approach to address a bleeding vessel or pedicle” (range of agreement, 28%-47%).

### Perceived Impact of Improved/Greater Articulation in Stapling on Surgical Access

Full results for statements related to the perceived impact of improved/greater articulation in stapling on surgical access are shown in **Table 4**. It should be noted that panelists’ responses were independent of observations made in scientific studies and represented their opinion.

Consensus opinion was reached that “greater jaw aperture feature could help to place thick or fragile tissues more easily in the stapler jaws” (consensus statement 2). Consensus was reached amongst the panelists that “the articulation of endocutters is extremely clinically important” to “[perform] a desired stapling job [in laparoscopic surgeries]” (consensus statement 3).

Panelists were in consensus that “improved access for such hard-to-reach targets and limited space during stapling would improve the safety of the surgery” (consensus statement 4) and “Endocutters with improved access through improved/greater articulation in stapling would become common use” (consensus statement 5).

Similar to the statements related to surgeon stress or concern, none of the subgroups reached agreement that the “inability of the device to access target anatomy” (range of agreement, 53%-75%) and “decreased visibility to access target anatomy becomes less stressful” (range of agreement, 55%-75%) if endocutters with improved/greater articulation was used.

Additionally, none of the subgroups were in agreement that 10% extra time [was spent on] pre-operative assessments for patients or procedures with predictable compromised surgical access (hard-to-reach targets and limited space) (range of agreement, 53%-78%).

Reduced unintentional tissue/structure damage (range of agreement, 83%-100%), reduced tension on the tissue/structure fired on (range of agreement, 88%-98%), reduced tearing of fragile tissue away from the staple line (range of agreement, 88%-96%), and reduced surgical stress (range of agreement, 83%-93%) also did not reach consensus.

**Specialty-specific differences**. It was agreed among colorectal and thoracic surgeons that improved access for hard-to-reach targets and limited space in stapling would reduce unintentional tissue/structure damage and would reduce surgical stress with the bariatric surgeon subgroup not reaching agreement on these statements (83% agreement for both statements).

Colorectal surgeons reached agreement on all 4 statements related to features that facilitate reaching difficult-to-access targets (**Table 4**). They agreed that having a greater jaw aperture, articulation span, and easy-to-use, one-handed operation would help surgeons to place thick or fragile tissues more easily in stapler jaws and improve access to targets.

However, colorectal surgeons were the only specialists who did not reach agreement that devices with improved access for hard-to-reach targets and limited space in stapling would reduce tension on the structure or tissue being fired on (88% agreement) and reduce tearing of fragile tissue away from the staple line (88% agreement). Across all 20 statements, the minimum level of agreement across surgical specialties was 35%.

**Region-specific differences.** Surgeons in Japan reached agreement on 3 out of 4 statements related to features that facilitate reaching difficult-to-access targets (**Table 4**). They were also in agreement that greater jaw aperture and articulation span would facilitate thick or fragile tissue placement in stapler jaws and improve access to targets.

However, there was least agreement (85% agreement) among panelists in Japan on whether surgeons would use endocutters, which gave improved access through greater articulation in place of the existing stapler choices. In contrast, surgeons in the US and Europe reached agreement on using endocutters with greater articulation (95% and 98% agreement, respectively).

Generally, there was agreement on statements related to the impact of devices that improved surgical access and reach across every region, with surgeons in Japan agreeing with all 5 statements, followed by the US reaching agreement on 4 out of 5 statements (**Table 4**). Surgeons in Europe agreed with only 3 out of 5 statements and did not reach agreement that devices with improved access reduced tearing of fragile tissue away from the staple line and reduced surgical stress (88% and 83% agreement, respectively). Across all 20 statements, the minimum level of agreement across regions was 35% (**Table 4**).

## DISCUSSION

Hard-to-reach targets and limited space for stapling present challenges for surgeons. While design improvements to endocutters seek to alleviate these constraints, the potential impact and unmet needs from surgeons’ perspective of limited surgical access and space on stapling have not yet been fully elucidated. This modified Delphi panel gained insights into surgeons’ perspective across a variety of specialties and from 3 distinct geographies.

Among the presented statements, consensus was achieved in areas pertaining to the impact of limited surgical access and space on surgical performance/outcome and the perceived impact of improved/greater articulation in stapling on surgical access. Specifically, panelists were in consensus about the clinical importance of articulation of endocutters (consensus statement 3). Given the restricted space in body cavities such as the pelvis and thorax, minimally invasive procedures in these areas are challenging and require the surgeon to navigate tight spaces.^12,23^ In this study, the panelists agreed that articulation of stapling devices is an important feature to achieve optimal stapling during laparoscopic surgery. Furthermore, the panelists’ consensus opinion was that improved access for hard-to-reach targets and limited space during stapling would improve safety of surgery (consensus statement 4). While comparative studies are limited, a wider range of articulation can permit difficult stapling angles and enables surgeons to access tissue in tight spaces, potentially mitigating the risk of instrument clashes.^24^

In thoracic surgeries, tissue slippage can also lead to complications, including but not limited to intraoperative bleeding.^18^ Similarly, in bariatric surgery, thick gastric tissue is susceptible to tissue slippage during stapling and may negatively impact staple line integrity.^13^ While the Delphi study panelists were in consensus that tissue slippage can occur in constricted anatomical spaces (consensus statement 1), tissue slippage can also be a challenge during bariatric surgeries which are performed in relatively less constricted spaces compared with the pelvic or thoracic cavity.^13^

Participants were also in consensus that greater jaw aperture would improve handling of thick or fragile tissues (consensus statement 2). A 2009 study by Gossot et al^19^ examined the occurrence of adverse events related to endoscopic stapler usage in VATS and drew similar conclusions. Possible underlying causes of surgical complications in VATS include the limited ability of staplers with a narrow jaw aperture in handling tissue, indicating how improvements to stapler design may improve outcomes in MIS.

By understanding user-specific challenges and needs from both specialty- and region-wide perspectives, endoscopic stapling devices can continue to be refined. In this study, improved articulation and greater jaw aperture were the key design features examined. In colorectal surgery, the tight space in the pelvis makes it technically challenging to perform anastomosis.^12^ Narrow confines of the pelvic cavity limits space for insertion of stapling devices, affecting tissue traction and optimal cutting angles. This may necessitate multiple linear staple firings which have been suggested to increase the risk of anastomotic leak.^25,26^ A stapler with improved articulation could potentially mitigate such risk, and offers insight as to why the colorectal surgeon panelists were largely in agreement on the features that could facilitate reaching difficult- to-access targets.

Bariatric panelists had a relatively lower level of agreement on the impact of limited surgical access and space on surgical performance/outcome(s) (minimum level of agreement remained at 81%), suggesting that they perhaps have greater ease in compensating for insufficient articulation and jaw aperture of endocutters compared with colorectal surgeons. It is possible that due to the relatively less constricted abdominal cavity, the optimal staple angle can be achieved with less articulation than required in a more constricted anatomical space.

From the perspective of thoracic surgeons, a 2018 study by Shimizu et al^27^ proposed that twisting and lifting actions exert stress on pulmonary vessels and decrease vessel stump endurance upon sealing. Such twisting and lifting actions constitute compensating behaviors performed by surgeons to reach hard-to-access targets with limited space for stapling, as surveyed in this Delphi panel study. Improved stapler articulation may limit the need for these compensating actions, increasing the likelihood of favorable surgical outcomes.

The survey responses gathered in this modified Delphi panel represented surgeon opinions gathered from a diverse range of regions and surgical specialties from a relatively large number of panelists. Although there was an expected drop-off in participation between survey rounds, this study was still able to facilitate the coverage of specialty-specific nuances, highlighting challenges that are more commonly faced in specific types of surgeries. Similarly, the Delphi study design enabled the gathering of country-specific challenges faced by surgeons and consolidation of a holistic view of the unmet needs in laparoscopic surgeries. Key among them is articulation of endocutters, as exemplified by the consensus opinion that endocutters with improved access through improved/greater articulation would become common use (consensus statement 5).

Nonetheless, this study does have limitations, attributed to the nature of Delphi studies. Consensus regarding the benefits of greater articulation and jaw aperture of endoscopic staplers was gathered by posing statements of a hypothetical nature. The study was also limited in gathering consensus on subjective behavioral traits such as stress and concern, as reflected by the lack of consensus in statements related to this area. The framing of these statements related to such subjective areas could be improved upon in future work to elicit a clearer response. Results were based on survey responses rather than real-world outcomes and should supplement evidence-based guidance, not replace it. In addition, more than 90% of the panelists were male, and therefore female surgeons are underrepresented in the current Delphi study.^28^ However, this study identified unmet needs with stapling devices during MIS on a region- and specialty-wide level from the perspective of the surgeon. Insights gathered from this study may lead to stapling device design improvements and potentially facilitate optimal surgical stapling in constricted anatomical spaces.

## CONCLUSION

While there is a growing body of literature on endoscopic stapling and studies investigating the usefulness of new stapler features, such studies tend to be conducted within specific countries and specialties. This Delphi panel provided a broader view of the unmet needs across specialties and countries from the perspective of the surgeon. Ultimately, the consensus opinion among participating surgeons was that endocutters with greater jaw aperture and articulation may improve surgical access and that such a device would become common use.

### Author Contributions

Substantial contributions to this study are as follows: conception and design: M.G., N.J., W.P., and S.R.; analysis and interpretation of the data: M.G., N.J., W.P., and S.R.; drafting the article or revising it critically for important intellectual content: M.G., N.J., W.P., and S.R.; and final approval of the version of the article to be published: M.G., N.J., W.P., and S.R.

### Disclosures

M.G., W.P., and S.R. are employees of Johnson & Johnson. N.J. was an employee of Johnson & Johnson at the time of the study.

## Supplementary Material

Online Supplementary Material Table 1. Expert Eligibility Criteria
Screening Questions
