# Supplementary material for: Global Surgeon Opinion on the Impact of Surgical Access When Using Endocutters Across Specialties
Source: J Health Econ Outcomes Res. 2023 Sep 20;10(2):62–71. doi: 10.36469/001c.87644 (PMC10515882; doi:10.36469/001c.87644)
Supplement: Online Supplementary Material — Table 1. Expert Eligibility Criteria Screening Questions [file jheor_2023_10_2_87644_180475.docx]

SUPPLEMENTAL MATERIALS

Table 1. Expert Eligibility Criteria

| **Specialty** | **Inclusion Criteria** |
| --- | --- |
| All | - 2 to 30 years post-residency - In practice full-time and ≥ 75% of time in direct patient care - Perform majority of procedures laparoscopically (summation of procedures performed via laparoscopy, hand-assisted laparoscopy, single incision laparoscopy, fully robotic and robotic hybrid procedures) - Perform ≥ 10 surgeries per month using endoscopic stapler - Be Board Certified if practicing in US - Not affiliated with any medical device company or serving as clinical investigator for any health care manufacturers - No one in their immediate family works for a health care product or medical device manufacturer, distributor or seller |
| Bariatric | - Bariatric surgery specialty or sub-specialty - Perform ≥ 6 sleeve gastrectomy/gastric bypass surgeries laparoscopically (summation of mini-thoracotomies, video-assisted thoracic surgeries, fully robotic and robotic hybrid procedures) per month on average - No more than 40% of bariatric surgeons are general surgeons |
| Colorectal | - Colorectal surgery specialty or sub-specialty - Perform ≥6 colorectal surgeries laparoscopically (summation of mini-thoracotomies, video-assisted thoracic surgeries, fully robotic and robotic hybrid procedures) per month on average, including right, transverse, left, sigmoid, total and/or proctocolectomy/low anterior resection |
| Thoracic | - Thoracic/cardiothoracic surgery specialty or sub-specialty - Perform ≥6 thoracic surgeries laparoscopically (summation of mini-thoracotomies, video-assisted thoracic surgeries, fully robotic and robotic hybrid procedures) per month on average, including lobectomy, wedge resection, pneumonectomy, segmentectomy |

**Screening Questions**

First, we would like you to answer several questions for background information. If you qualify, we will invite you to participate in this research study.

SX. Record Sex

1 Male

2 Female

S1. Which one of the following best describes your specialty? *(Record one response.)*

1. General Surgery

2 Colorectal Surgery

3 Bariatric Surgery

4 Thoracic/Cardiothoracic Surgery

5 Other (specify:___________________) 🡪 **TERMINATE**

S1b. What is your surgical sub-specialty? *(Record one response.)*

2 Colorectal Surgery

3 Bariatric Surgery

4 Thoracic/Cardiothoracic Surgery

7 Other (specify:___________________) 🡪 **TERMINATE**

8 Do not have a surgical sub-specialty 🡪 **TERMINATE**

**US ONLY**

S2. Are you board certified in **[ANSWER FROM S1]**?

1 Yes

2 No 🡪 **TERMINATE IF GENERAL SURGEON; OTHERWISE CONTINUE.**

S3. In total, how many years have you been in practice, post residency?

_______ Years **MUST BE 2 TO 30 YEARS. OTHERWISE, TERMINATE.**

S4. Are you in practice…?

1 Full time

2 Part Time 🡪 **TERMINATE**

S5. What percentage of your time is devoted to clinical practice, rather than research, teaching or administration duties? **MUST ADD TO 100%.**

_______% treating patients

_______% in academic or administrative setting 🡪 **TERMINATE IF > 50%**

S6. Which of the following best describes the primary hospital/facility in which you operate?  *(Select one response.)*

1. Teaching Hospital, either public or private; (i.e. medical school, university affiliated, etc.)
2. Non-teaching Hospital (i.e. city, county, community or state hospital, privately-owned, corporately-owned, etc.)
3. Veterans Administration
4. Military Hospital
5. Ambulatory Surgery Center – Hospital owned
6. Ambulatory Surgery Center - Free-standing/Non-hospital owned
7. Clinic
8. Other (Specify) _____________

S7. In an average month, please indicate the **TOTAL** number of surgeries you, yourself, perform.

___________ Procedures per month
**[RECORD EXACT. TERMINATE IF <10. RANGE 0-400.]**

S8. Of the total number of procedures you perform in an average month, for what percentage are each of the following techniques used? **(Please enter a whole number for each. Total must equal 100%)**

**BARIATRIC (GASTRIC) / COLORECTAL / GENERAL SURGEONS SHOW:**

| _____% | Open |
| --- | --- |
| _____% | Laparoscopic |
| _____% | Hand-assisted (HALS) |
| _____% | Single Incision Laparoscopy (SILS) - sometimes called SSL, SILS, or LESS |
| _____% | Fully robotic (Using the robotic system/tools to perform nearly all of key procedure steps from the surgeon console) |
| _____% | Robotic Hybrid (Using a mix of a robotic system/tools, along with laparoscopic tools to perform the key procedure steps) |
| **MUST = 100%** |  |

***NOTE: SURGEONS MUST PERFORM LAPAROSCOPIC + HALS + SILS + ROBOTICS FOR THE MAJORITY OF THEIR PROCEDURES TO CONTINUE. IF OPEN IS HIGHER, TERMINATE.***

**THORACIC SURGEONS SHOW:**

| _____% | Open (large incision with rib spreading) |
| --- | --- |
| _____% | Mini-Thoracotomy (smaller incisions, no rib spreading) |
| _____% | VATS (thoracoscopy, port access) |
| _____% | Fully robotic (Using the robotic system/tools to perform nearly all of key procedure steps from the surgeon console) |
| _____% | Robotic Hybrid (Using a mix of a robotic system/tools, along with laparoscopic tools to perform the key procedure steps) |
| **MUST = 100%** |  |

***NOTE: SURGEONS MUST PERFORM MINI-THORACOTOMY + VATS + ROBOTIC FOR THE MAJORITY OF THEIR PROCEDURES TO CONTINUE. IF ANOTHER TECHNIQUE IS HIGHER, TERMINATE.***

S9. Of the <**insert total # from the calculation S7 x S8 % laparoscopic/minimally invasive**> laparoscopic (thoracoscopic)/minimally invasive procedures you perform in an average month, approximately how many are for each of the following procedures you perform as the primary surgeon? *(Enter a number for each procedure listed.)*

**COLORECTAL: MUST PERFORM 10+ PROCEDURES, 6+ MUST BE LAPAROSCOPIC COLORECTAL PROCEDURES (RIGHT, TRANSVERSE, LEFT, SIGMOID, TOTAL AND/OR PROCTOCOLOCTOMY/LOW ANTERIOR RESECTION)**

|  | **Total # Laparoscopic/Minimally Invasive Procedures**  **Per Month** |
| --- | --- |
| **SHOW IF GENERAL SURGEON** |  |
| Appendectomy | **_____** |
| Cholecystectomy | **_____** |
| Inguinal Hernia | **_____** |
| Ventral Hernia | **_____** |
| Nissen Fundoplication | **_____** |
| Esophagectomy | **_____** |
| Splenectomy | **_____** |
| Other General Procedures | **_____** |
| **SHOW IF COLORECTAL OR GENERAL SURGEON** | **_____** |
| Total Colectomy | **_____** |
| Right Colectomy / hemicolectomy |  |
| Transverse Colectomy | **_____** |
| Left Colectomy / hemicolectomy | **_____** |
| Sigmoidectomy | **_____** |
| Low Anterior Resection | **_____** |
| Other Colorectal Procedures | **_____** |
| **SHOW IF BARIATRIC (GASTRIC) OR GENERAL SURGEON** |  |
| **[US/Europe only]** Gastric Bypass | **_____** |
| **[US/Europe only]** Gastric Banding | **_____** |
| **[US/Europe only]** Sleeve Gastrectomy | **_____** |
| **[US/Europe only]** Duodenal Switch |  |
| **[US/Europe only]** Gastrectomy |  |
| **[US/Europe only]** Other Bariatric procedures | **_____** |
| **[APAC only]** Other Gastric Procedures |  |
| **SHOW IF THORACIC OR CARDIO THORACIC SURGEON** |  |
| Lobectomy | **_____**  **THORACIC/CARDIO THORACIC: MUST PERFORM 10+ PROCEDURES OF WHICH 6+ ARE LOBECTOMY, WEDGE RESECTION, PNEUMONECTOMY, SEGMENTECTOMY)** |
| Wedge resection | **_____** |
| Pneumonectomy | **_____** |
| Esophagectomy | **_____** |
| Segmentectomy | **_____** |
| Pleural adhesions | **_____** |
| Other Thoracic/Cardiothoracic Procedures | **_____** |
| **SHOW FOR ALL SURGEONS** |  |
| Other Procedures | **_____** |
| Total | **MUST BE EQUAL TO CALCULATION**  **(S7# XS8%)** |

***NOTE: GENERAL SURGEONS MAY QUALIFY AS COLORECTAL OR BARIATRIC IF MEET PROCEDURE REQUIREMENT. NO MORE THAN 40% OF COLORECTAL OR BARIATRIC MAY BE GENERAL SURGEONS.***

**BARIATRIC (US/EMEA): MUST PERFORM 10+ PROCEDURES OF WHICH 6+ ARE LAPAROSCOPIC SLEEVE GASTRECTOMY OR GASTRIC BYPASS**

**GASTRIC (JAPAN): MUST PERFORM 10+ PROCEDURES OF WHICH 6+ ARE LAPAROSCOPIC GASTRECTOMIES.**

S10. In an average month, approximately how many **[INSERT: colorectal, bariatric or thoracic]** procedures do you perform in which you use endoscopic linear stapling device, such as (EndoGIA or Echelon)?

________ # Procedures per month use an **endoscopic linear stapling device**

**MUST PERFORM AT LEAST 10 PROCEDURES PER MONTH USING AN ENDOSCOPIC LINEAR STAPLING DEVICE.**

S11. Are you, yourself, currently affiliated with any medical device company or other health care manufacturer serving as a clinical investigator, consultant, researcher or any other capacity? *(Select one response.)*

1 Yes 🡪 **TERMINATE**

2 No

S12. Do you or does anyone in your immediate family work for any of the following businesses? *(Select one response.)*

1 A company that manufactures, distributes or sells health care products 🡪 **TERMINATE**

2 A medical device manufacturer or distributor 🡪 **TERMINATE**

3 None of these

**Round 1 Survey Questions**

Q1. When using an **endoscopic linear stapling device** (such as an EndoGIA or Echelon), which of the following PROBLEMS, if any, could occur due to hard-to-reach targets and/or limited space for stapling. *(Select all that apply.)*

**RANDOMIZE LIST**

1. Unintentional tissue damage while preparing the place to put the anvil
2. Unintentional tissue damage while placing the anvil under the tissue/vessel
3. Unintentional tissue damage while firing the stapler
4. Tearing of fragile tissue away from the staple line
5. Tissue slippage outside of the jaws while transecting
6. Unintended distal tip movement while firing the stapler
7. Need to use two hands, making it challenging to manage other devices
8. Need extra reloads to compensate for tissue slipping outside the jaws
9. Tearing of fragile tissue along the staple line
10. Damage of an important structure I am unable to see while firing
11. Increased tension on the structure or tissue I am firing on
12. Poor staple line quality
13. Insufficient transection
14. Lose exposure to targeted tissue
15. Retracing of steps has potential to tear or traumatize tissue
16. Inadequate margin
17. Problematic perpendicular transection
18. Insufficient stricture
19. None of the above

Q1b. In what percentage of the laparoscopic (**THORACICS:** thoracoscopic) procedures you perform in an average month does one or more of these problems occur?

________ % laparoscopic (thoracoscopic) procedures where one or more of these problems occur

Q2. Which of the following **complications** of surgery could result due to hard-to-reach targets and limited space while using the endoscopic linear stapling device to create a staple line? *(Select all that apply.)*

**RANDOMIZE LIST**

1. Staple line oozing/bleeding controllable with clips, suture or fibrin glue
2. Staple line oozing that requires 15 minutes or more of additional operating room time
3. Bleeding that requires conversion to open
4. Tissue trauma that requires further repair
5. Unintentional tissue/structure damage
6. Tearing of fragile tissue away from the staple line
7. Tearing of fragile tissue along staple line
8. Tension on the structure or tissue firing on
9. Less surgical margin than expected
10. Perpendicular transection not possible/easy
11. Life threatening, permanently disabling, or fatal complications
12. Stenosis
13. Other (Specify) _______________
14. None of the above

Q3. Thinking about when you are stapling with an **endoscopic linear stapling** **device** during surgery. What are all the types of compensating behaviors you adopt to and/or adjust for in hard to reach targets and limited? *(Please be as detailed as possible in your response.)*

| **CAPTURE OPEN-ENDED RESPONSE.** |
| --- |

Q4. Thinking of problematic procedures due to hard-to-reach targets and limited space, what percentage of those procedures in an average month does an intraoperative complication become a cause of a post-operative complication? (Record 0-100%)

**_____ % of problematic procedures in an average month does an intraoperative complication become cause of post-operative complication**

**Now we would like to ask you a few questions about the impact of the device on your stress and concern during situations with compromised surgical access (hard to reach targets and limited space).**

Q5. Do you experience stress or concern when you have a less accessible approach to a vessel or pedicle that may increase the chance of harm/injury to a vessel or pedicle?

1 Yes

2 No

Q6. Do you experience physical distress or discomfort when facing a situation of a hard-to-reach area with difficult approach to address a bleeding vessel or pedicle?

1 Yes

2 No

Q7. Do you experience stress or concern that a hard-to-reach bleeding pedicle or vessel that may lead to a higher probability of surgical complication?

1 Yes

2 No

Q8. Do you experience stress or concern that a hard-to-reach area that may compromise your surgical training during a procedure?

1 Yes

2 No

Q9. Do you experience stress or concern when you must adjust to the limits of the device?  *(Select one response.)*

1 Yes

2 No

Q10. Do you anticipate that you would have less stress or concern if you were using an endoscopic linear stapling device with improved articulation in surgery?

1 Yes

2 No

Q11. Do you anticipate that you would have less stress or concern if you were training a fellow or resident during surgery with an endoscopic linear stapling device with improved articulation?

1 Yes

2 No

**Now we would like to think about when you are stapling in hard-to-reach targets and limited space during Laparoscopic, Open and Robotic Surgeries.**

Q12a. Do you experience stress or concern when you need to convert from laparoscopic to open surgery due to failure of an endoscopic linear stapler to secure pedicle or blood vessel?  *(Select one response.)*

1 Yes

2 No

Q12b. In what percentage of the **[INSERT: colorectal, bariatric or thoracic]** procedures you perform on a monthly basis do you need to convert from laparoscopic to open surgery due to failure of an endoscopic linear stapler to secure pedicle or blood vessel? *(Record 0-100%)*

_____ % need to convert from laparoscopic to open

Q13. Assuming you were performing surgery with an endoscopic linear stapling device with improved articulation in stapling, which of the following potential issues related to laparoscopic surgery would be less stressful? *(Select all that apply.)*

1. Space restrictions
2. Difficult angles
3. Having to work adjacent to critical structures
4. Decreased visibility
5. Inability of device to access target
6. Dealing with compromised (or diseased) tissue
7. Other (Specify) ____________

Q14. Now we would like you to rank order the features listed below in terms of which could help you reach difficult to access targets. To do this, please place a “1” next to the feature that would help you the most, place a “2” next to the feature that would help you the second most and continue until all features are ranked.

| **RANDOMIZE LIST** | **Rank 1-5** |
| --- | --- |
| Greater articulation span |  |
| Powered articulation |  |
| Ability to stop the device jaw at any point/increment along the articulation span |  |
| Greater jaw aperture |  |
| Easy to use, one-handed operation |  |

**ASK Q15 IF S8 ROBOTIC IS 1% OR MORE, OTHERWISE SKIP TO NEXT QUESTION.**

Q15. Do you anticipate that you would have less stress or concern when an assistant is firing an **endoscopic linear stapling device with improved articulation** during a **robot-assisted surgery** procedure where there may be hard to reach targets and limited space increasing the difficulty of firing? *(Select one response.)*

1 Yes

2 No

**Now we would like talk about the clinical importance of ACCESS in stapling.**

Q16a. In a procedure with predictable compromised surgical access (hard to reach targets and limited space), Do you typically spend extra time in pre-operative assessment? *(Select one response.)*

1 Yes

2 No **– SKIP TO Q17**

Q16b. Again, thinking about the procedures with predictable compromised surgical access (hard to reach targets and limited space), in what percentage of your cases do you need to spend extra time in pre-operative assessment? *(Record 0-100%)*

_____ % predictable compromised surgical access cases you spend extra time in pre-operative assessments

Q17. For each of the following, please indicate how **clinically** **important** articulation of an endoscopic linear stapling device is to you with respect to performing your desired stapling job.

| **[DO NOT SHOW SCALE NUMBERS]** | **Extremely Clinically Important** | **Very Clinically Important** | **Somewhat Clinically Important** | **Not Very Clinically Important** | **Not at All Clinically Important** |
| --- | --- | --- | --- | --- | --- |
| Surgery in general | 5 | 4 | 3 | 2 | 1 |
| Open surgery | 5 | 4 | 3 | 2 | 1 |
| Laparoscopic surgery | 5 | 4 | 3 | 2 | 1 |

**Now we would like to talk about clinical importance of IMPROVED ACCESS in stapling.**

**“Improved” access in stapling is the ability of the surgeon to use the endoscopic linear stapler to access hard to reach targets and limited space, exactly as intended for formation of the staple lines.**

Q20. Overall, do you perceive that **improved** **access** through improved **articulation** in stapling would have a positive clinical effect on surgical outcomes?

1 Yes

2 No

Q21. Which, if any, of the following improvements would you expect **improved access** to offer for hard-to-reach targets and limited space during stapling? *(Select all that apply.)*

1. Reduced unintentional tissue / structure damage
2. Reduced tearing of fragile tissue away from the staple line
3. Reduced tearing of fragile tissue along the staple line
4. Reduced tension on the structure or tissue I’m firing on
5. Reduced staple line oozing / bleeding
6. Reduced bleeding resulting from reduced damage to tissue / structure
7. Improved staple formation
8. Improved staple line quality / integrity
9. Reduced the risk not to keep the appropriate surgical margin
10. Decreased trauma to the surrounding tissue / anatomical structures
11. Improved user experience
12. Reduced surgery stress
13. Peace of mind
14. Improved safety of surgery
15. Reduced rate of hemorrhage
16. Other (Specify) ________________
17. None of the above

Q22. If an endoscopic linear stapler gave you **improved access** through **improved articulation** in stapling compared to currently available choices, how often would you expect to use it in your procedures, assuming this device is available where you practice? *(Select one answer.)*

| I would use in all of my procedures | 1 |
| --- | --- |
| I would use in most of my procedures | 2 |
| I would use in some of my procedures | 3 |
| I would use in a few of my procedures | 4 |
| I would not use in any of my procedures | 5 |

Q23. Do you expect an endoscopic linear stapler with **improved access** through **improved articulation** to be widely used by surgeons?

1 Yes

2 No

Q24. Would an endoscopic linear stapler with **improved access** through **improved articulation** in stapling be a standard of care?

1 Yes

2 No

**Classification Questions**

D1. Which of the following best describes the setting in which you practice? *(Select one response.)*

1 Urban

2 Suburban

3 Rural

D2. Approximately how many beds are in the hospital with which you are primarily affiliated? **Min=1; Max=9999**

_______ **[RECORD EXACT.]**

D3. Approximately how many OR suites are in the hospital with which you are primarily affiliated? **Min=1; Max=9999**

_______ **[RECORD EXACT.]**

D4. Are you employed by the hospital or in a private practice? *(Select one response.)*

1. Hospital-Based
2. Private Practice
3. Other (specify):________________________________

If we missed anything, please mention that below.

**CAPTURE OPEN-ENDED RESPONSE.**

**Round 2 Questions**

1. Thinking about when you are using an **endoscopic linear stapling device** (such as: EndoGIA or Echelon, please indicate whether you **agree or disagree** with each statement listed below? (Select one answer for each statement.)

| **[RANDOMIZE]** | **Agree** | **Disagree** |
| --- | --- | --- |
| Tearing of fragile tissue along the staple line can occur due to hard-to-reach targets and/or limited space for stapling | 2 | 1 |
| Needing extra reloads to compensate for tissue slipping outside the jaws can occur due to hard-to-reach targets and/or limited space for stapling | 2 | 1 |
| Increased tension on the structure or tissue I am firing on can occur due to hard-to-reach targets and/or limited space for stapling | 2 | 1 |
| Tissue slippage outside of the jaws while transecting can occur due to hard-to-reach targets and/or limited space for stapling | 2 | 1 |
| Tearing of fragile tissue away from the staple line can occur due to hard-to-reach targets and/or limited space for stapling | 2 | 1 |
| Poor staple line quality can occur due to hard-to-reach targets and/or limited space for stapling | 2 | 1 |

1. And, when you are stapling with an endoscopic linear stapling device **during surgery**, please indicate whether you **agree or disagree** with each statement listed below? (Select one answer for each statement.)

| **[RANDOMIZE]** | **Agree** | **Disagree** |
| --- | --- | --- |
| Tearing of fragile tissue along the staple line can occur due to hard-to-reach targets and/or limited space for stapling | 2 | 1 |
| Less surgical margin than expected can result as a complication of surgery due to hard-to-reach targets and limited space | 2 | 1 |
| Staple line oozing/bleeding controllable with clips, suture, or fibrin glue can result as a complication of surgery due to hard-to-reach targets and limited space | 2 | 1 |
| Tissue trauma that requires further repair can result as a complication of surgery due to hard-to-reach targets and limited space | 2 | 1 |
| Tearing of fragile tissue away from the staple line can result as a complication of surgery due to hard-to-reach targets and limited space | 2 | 1 |

1. For each of the following statements, please indicate whether you **agree or disagree** with each. (Select one answer for each statement.)

| **[RANDOMIZE]** | **Agree** | **Disagree** |
| --- | --- | --- |
| Additional/further dissection is the type of compensating behavior I adopt to and/or adjust for in hard-to-reach targets and limited space. | 2 | 1 |
| Adding or using another port is the type of compensating behavior I adopt to and/or adjust for in hard-to-reach targets and limited space | 2 | 1 |
| The use of different staples/different staple loads is the type of compensating behavior I adopt to and/or adjust for in hard-to-reach targets and limited space. | 2 | 1 |
| Angulation change/change angle of approach/twist/rotate/move around is the type of compensating behavior I adopt to and/or adjust for in hard-to-reach targets and limited space. | 2 | 1 |
| Visualization change/look from different perspectives is the type of compensating behavior I adopt to and/or adjust for in hard-to-reach targets and limited space. | 2 | 1 |
| Suturing over trouble spots is the type of compensating behavior I adopt to and/or adjust for in hard-to-reach targets and limited space. | 2 | 1 |
| The use of multiple fires/double stapling is the type of compensating behavior I adopt to and/or adjust for in hard-to-reach targets and limited space. | 2 | 1 |
| The use of staple line reinforcing is the type of compensating behavior I adopt to and/or adjust for in hard-to-reach targets and limited space. | 2 | 1 |
| The use of additional trocars/ switch or change trocars is the type of compensating behavior I adopt to and/or adjust for in hard-to-reach targets and limited space. | 2 | 1 |
| Delicate/slower/more fine handling of stapler is the type of compensating behavior I adopt to and/or adjust for in hard-to-reach targets and limited space. | 2 | 1 |

1. For each of the following statements, please indicate whether you **agree or disagree** with each. (Select one answer for each statement.)

| **[RANDOMIZE]** | **Agree** | **Disagree** |
| --- | --- | --- |
| I experience stress or concern when I have a less accessible approach to a vessel or pedicle that may increase the chance of harm/injury to a vessel or pedicle. | 2 | 1 |
| I do not experience physical distress or discomfort when facing a situation of a hard-to-reach area with difficult approach to address a bleeding vessel or pedicle | 2 | 1 |
| I experience stress or concern that a hard-to-reach bleeding pedicle or vessel may lead to a higher probability of surgical complication. | 2 | 1 |
| I do not experience stress or concern that a hard-to-reach area may compromise my surgical training during a procedure. | 2 | 1 |
| I experience stress or concern when I must adjust to the limits of the device. | 2 | 1 |
| I anticipate that I would have less stress or concern if I was using an endoscopic linear stapling device with improved articulation in surgery. | 2 | 1 |
| I anticipate that I would have less stress or concern if I was training a fellow or resident during surgery with an endoscopic linear stapling device with improved articulation. | 2 | 1 |
| I experience stress or concern when I need to convert from laparoscopic to open surgery due to failure of an endoscopic linear stapler to secure pedicle or blood vessel. | 2 | 1 |

1. Thinking about when you are performing laparoscopic surgery using an endoscopic linear stapler device **with improved/greater articulation in stapling**, please indicate whether you **agree or disagree** with each of the statements listed below? (Select one answer for each statement.)

| **[RANDOMIZE]** | **Agree** | **Disagree** |
| --- | --- | --- |
| Difficult angles become less stressful. | 2 | 1 |
| Inability of the device to access target anatomy becomes less stressful. | 2 | 1 |
| Space restrictions become less stressful. | 2 | 1 |
| Decreased visibility to access target anatomy becomes less stressful. | 2 | 1 |

1. And thinking of a **new stapler**, please indicate whether you **agree or disagree** with each of the statements listed below? (Select one answer for each statement.)

| **[RANDOMIZE]** | **Agree** | **Disagree** |
| --- | --- | --- |
| Easy to use, one-handed operation feature could help me reach difficult to access targets. | 2 | 1 |
| Greater articulation span feature could help me reach difficult to access targets. | 2 | 1 |
| Greater jaw aperture feature could help me reach difficult to access targets. | 2 | 1 |
| Greater jaw aperture feature could help to place thick or fragile tissue more easily in the stapler jaws. | 2 | 1 |

1. And again, for each of the following statement listed below, please indicate whether you **agree or disagree** with each. (Select one answer for each statement.)

| **[RANDOMIZE]** | **Agree** | **Disagree** |
| --- | --- | --- |
| I have less stress or concern when an assistant is firing an endoscopic linear stapling device with improved articulation during a robot-assisted surgery procedure, where there may be hard to reach targets and limited space increasing the difficulty of firing. | 2 | 1 |
| I do not spend extra time in pre-operative assessment for a procedure with predictable compromised surgical access (hard-to-reach targets and limited space). | 2 | 1 |
| I spend 10% extra time in pre-operative assessment for patients with predictable compromised surgical access (hard-to-reach targets and limited space). | 2 | 1 |
| When performing a desired stapling job, the articulation of an endoscopic linear stapling device is extremely clinically important for laparoscopic surgeries. | 2 | 1 |
| I perceive that improved access through improved/greater articulation in stapling would have a positive clinical effect on surgical outcomes. | 2 | 1 |

1. For each statement listed below, please indicate whether you agree or disagree that you expect improved access for hard-to-reach targets and limited space during stapling would….? (Select one answer for each statement.)

| **[RANDOMIZE]** | **Agree** | **Disagree** |
| --- | --- | --- |
| Improve safety of surgery | 2 | 1 |
| Reduce surgical stress | 2 | 1 |
| Reduce tearing of fragile tissue away from the staple line | 2 | 1 |
| Reduce unintentional tissue/ structure damage | 2 | 1 |
| Reduce tension on the structure or tissue I’m firing on | 2 | 1 |

1. For each statement listed below, please indicate whether you agree or disagree that you expect improved access for hard-to-reach targets and limited space during stapling would….? (Select one answer for each statement.)

| **[RANDOMIZE]** | **Agree** | **Disagree** |
| --- | --- | --- |
| If an endoscopic linear stapler gave improved access through improved/greater articulation in stapling compared to currently available choices, I would use in most of my procedures | 2 | 1 |
| An endoscopic linear stapler with improved access through improved/greater articulation in stapling would become standard of care. | 2 | 1 |
